# Supplementary material for: Dysregulated lactate metabolism synergizes with ALS genetic risk factors to accelerate motor decline
Source: bioRxiv. 2025 Nov 25:2025.11.24.690227. Preprint. [Version 1] doi: 10.1101/2025.11.24.690227 (PMC12697354; doi:10.1101/2025.11.24.690227)

bioRxiv preprint doi: https://doi.org/10.1101/2025.11.24.690227; this version posted November 25, 2025. The copyright holder for this preprint (which was not certified by peer review) is the author/funder, who has granted bioRxiv a license to display the preprint in perpetuity. It is made available under aCC-BY 4.0 International license.

Supplementary Figure 1. Density of normal and degenerating axons in tibial nerves of WT, TDP43$^{Q331K/+}$, LDHB MNKO, and LDHB MNKO; TDP43$^{Q331K/+}$ mice **(A & B).** Representative images of 63x toluidine blue-stained sections of Femoral nerves from 3-month-old TDP43$^{Q331K/+}$ and LDHB MNKO; TDP43$^{Q331K/+}$ mice **(C & D).** Latency to fall from an inverted screen in 6-month-old animals **(E).** ***p<0.001, **p<0.01.

bioRxiv preprint doi: https://doi.org/10.1101/2025.11.24.690227; this version posted November 25, 2025. The copyright holder for this preprint (which was not certified by peer review) is the author/funder, who has granted bioRxiv a license to display the preprint in perpetuity. It is made available under aCC-BY 4.0 International license.

Figure S1

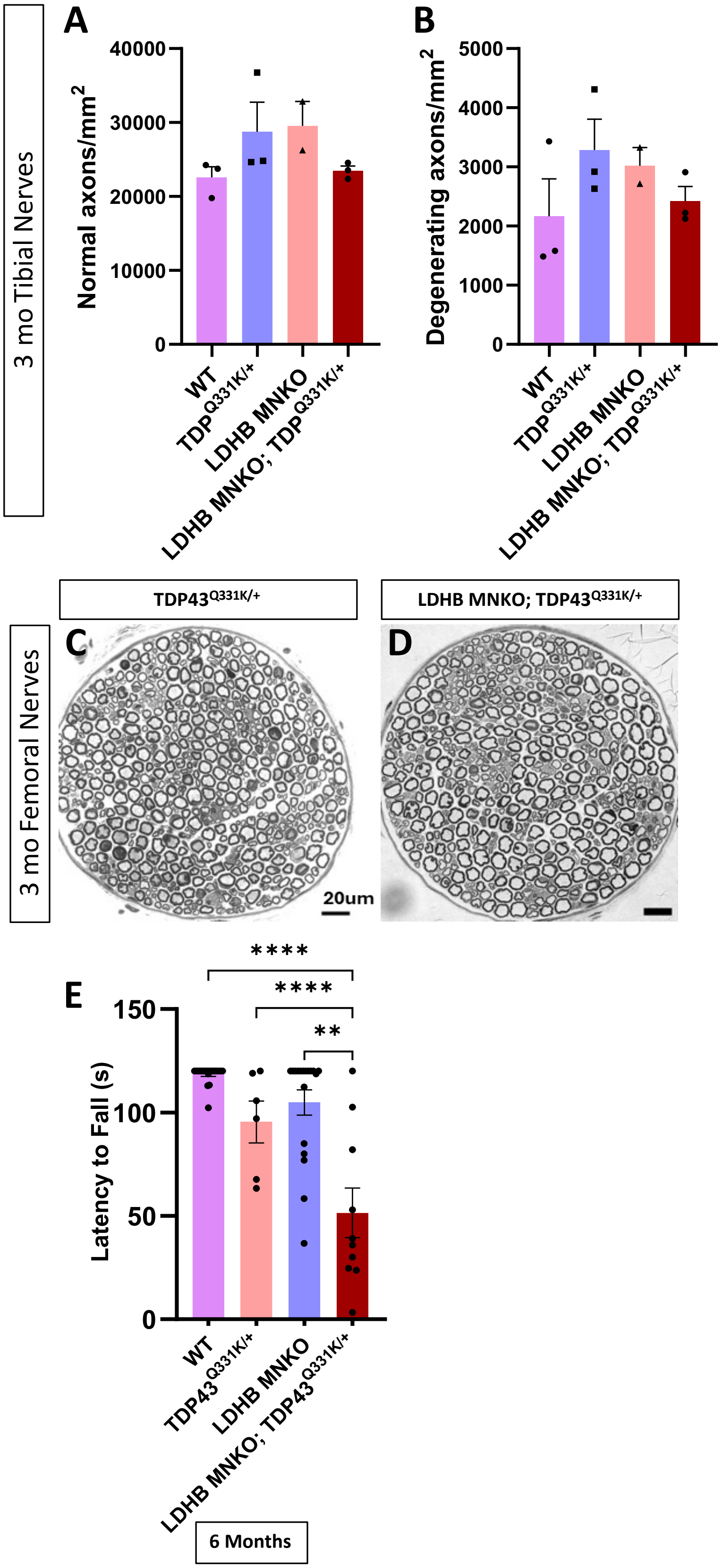

Supplement: Supplement 1 [file NIHPP2025.11.24.690227v1-supplement-1.pdf]
